# Natural HDAC‐1/8 inhibitor baicalein exerts therapeutic effect in CBF‐AML

**DOI:** 10.1002/ctm2.154

**Published:** 2020-08-26

**Authors:** Xiaoxuan Yu, Hui Li, Po Hu, Yingjie Qing, Xiangyuan Wang, Mengyuan Zhu, Hongzheng Wang, Zhanyu Wang, Jingyan Xu, Qinglong Guo, Hui Hui

**Affiliations:** ^1^ State Key Laboratory of Natural Medicines Jiangsu Key Laboratory of Carcinogenesis and Intervention Key Laboratory of Drug Quality Control and Pharmacovigilance Ministry of Education Jiangsu Key Laboratory of Drug Design and Optimization China Pharmaceutical University China Pharmaceutical University Nanjing Jiangsu China; ^2^ Department of Pharmacology School of medicine & Holostic integrative medicine Nanjing University of Chinese Medicine Nanjing Jiangsu China; ^3^ Department of Hematology The Affiliated DrumTower Hospital of Nanjing University Medical School Nanjing China

**Keywords:** ABC transporter genes, CBF‐AML, differentiation, HDAC‐1/8 inhibitors, natural product

## Abstract

**Background:**

Although targeting histone deacetylases (HDACs) may be an effective strategy for core binding factor‐acute myeloid leukemia (CBF‐AML) harboring t(8;21) or inv(16), HDAC inhibitors are reported to be limited by drug‐resistant characteristic. Our purpose is to evaluate the anti‐leukemia effects of Baicalein on CBF‐AML and clarify its underlying mechanism.

**Methods:**

Enzyme activity assay was used to measure the activity inhibition of HDACs. Rhodamine123 and RT‐qPCR were employed to evaluate the distribution of drugs and the change of ATP‐binding cassette (ABC) transporter genes. CCK8, Annexin V/PI, and FACS staining certified the effects of Baicalein on cell growth, apoptosis, and differentiation. Duolink and IP assay assessed the interaction between HDAC‐1 and ubiquitin, HSP90 and AML1‐ETO, and Ac‐p53 and CBFβ‐MYH11. AML cell lines and primary AML cells‐bearing NOD/SCID mice models were used to evaluate the anti‐leukemic efficiency and potential mechanism of Baicalein in vivo.

**Results:**

Baicalein showed HDAC‐1/8 inhibition to trigger growth suppression and differentiation induction of AML cell lines and primary AML cells. Although the inhibitory action on HDAC‐1 was mild, Baicalein could induce the degradation of HDAC‐1 via ubiquitin proteasome pathway, thereby upregulating the acetylation of Histone H3 without promoting ABC transporter genes expression. Meanwhile, Baicalein increased the acetylation of HSP90 and lessened its connection to AML1/ETO, consequently leading to degradation of AML1‐ETO in t(8;21)q(22;22) AML cells. In inv(16) AML cells, Baicalein possessed the capacity of apoptosis induction accompanied with p53‐mediated apoptosis genes expression. Moreover, CBFβ‐MYH11‐bound p53 acetylation was restored via HDAC‐8 inhibition induced by Baicalein contributing the diminishing of survival of CD34^+^ inv(16) AML cells.

**Conclusions:**

These findings improved the understanding of the epigenetic regulation of Baicalein, and warrant therapeutic potential of Baicalein for CBF‐AML.

## Graphical abstract

Baicalein shows HDAC1/8 inhibition without promoting ABC transporter genes expression.2. Baicalein exerts therapeutic effect in CBF‐AML.3. The inhibition of HDAC1/8 could be potential therapeutic target for CBF‐AML treatment.

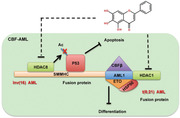

**Abbreviations**
AML: acute myeloid leukemia
CBF: core binding factor
CHX: cyclohexumide
CM: CBFβ‐SMMHC
CTCL: cutaneous T‐cell lymphoma
DMSO: dimethylsulfoxide
FAB: French‐American‐British
FITC: fluorescein isothiocyanate
HDAC: histone deacetylase
HSP90: core binding factor
LSC: leukemia stem cell
MDR1: multidrug resistance 1
NAB: sodium butyrate
N‐CoR: nuclear receptor corepressor
PE: phycoerythrin
SMMHC: smooth muscle myosin heavy chain

## 1 INTRODUCTION

Acute myeloid leukemia (AML) is an aggressive hematological neoplasm caused by abnormal hematopoietic progenitor cells.^1^, ^2^ Most of AML patients harbor nonrandom and somatically acquired chromosomal aberrations including but not limited to inversion, insertions, deletion, trisomy, monosomy, and reciprocal cytogenetic translocation.^3^ The core binding factor AML (CBF‐AML) consists of 15% of adult AML and 30% of childhood AML, which has a favorable prognosis but the 5‐year survival rate remains low.^4^

CBF‐AML encodes two recurrent cytogenetic abnormalities referred to as t(8; 21)q(22;22) and inv (16).^5^ The t(8; 21)q(22;22) is a common translocation identified in 40‐50% of FAB‐M2b subtype, and rare cases of M0, M1, and M4 subtypes.^6^ Meanwhile, the inv(16) occurs in 5% of AML cases.^7^ The abnormality of inv(16) is highly correlated with the AML subtype FAB‐M4 with dysplastic eosinophils in bone marrow (M4E_0_).^8^, ^9^ The CBFs consists of one CBF‐β subunit and three possible CBF‐α subunits, which are a group of DNA‐binding transcription factors.^10^ However, the fusion of *AML1*, whose encoding protein is a subunit of CBF‐α, and *ETO* generates a novel gene *AML1‐ETO*, resulting in a lack of the carboxyl terminal transactivation domain of *AML1*, which suggests that the *AML1‐ETO* disrupts hematopoiesis through a dominant‐negative mechanism.^11^ The ETO recruits histone deacetylase (HDAC) and associates with nuclear receptor corepressor (N‐CoR) that acts to repress the transcription of AML1 target genes.^12^ Evidence show that the degradation of the AML1‐ETO fusion protein is a target of t(8; 21)q(22;22) AML, and AML‐ETO is a client protein of HSP90 reducing the stability of AML1‐ETO and causing its degradation.^13^

In the other type of CBF‐AML, the inv(16) results in the fusion of *CBF‐β* with *MYH11* gene. The two non‐ampliflying inv(16) cases form two chimeric genes, *CBF/MYH11* and *MYH11/CBF*. However, only *CBFβ/MYH11* that encodes a CBFβ‐MYH11 smooth muscle myosin heavy chain (SMMHC) protein contributes to the leukemogenesis.^14^ Similar to AML1‐ETO, the CBFβ‐SMMHC (CM) form co‐repressor complexes, leading to recruitment of HDACs and silence target genes.^15^

Interfering with the function of pro‐leukemic fusion proteins is an effective strategy for AML treatment. HDACs are critical epigenetic modulating‐factors implicated in cancer, especially in causation and progression of CBF‐AML.^16^, ^17^ The two types of fusion proteins in CBF‐AML are both capable of recruiting HDACs, thus resulting in repression of target genes. HDAC inhibitors influence genes involved in cell differentiation, proliferation, and survival. The expression of HDAC‐1 is negative correlated with the prognosis and a specific target for inhibiting cell proliferation and leading to terminal differentiation in AML.^18^, ^19^ As a substrate of HDAC‐1, HSP90 can be inhibited through acetylation on lysine residues by HDAC‐1.^20^ HDAC‐8 is another class I HDAC that has been reported to be overexpressed in neuroblastoma, glioma, childhood acute lymphoblastic leukemia, and T‐cell lymphoma.^21^, ^22^, ^23^ HDAC‐8 has been shown to interact with the CM chimeric protein and to impair acetylation and inactivation of p53 that bound to CM, thus promoting CM‐associated leukemia stem cell (LSC) transformation and maintenance.^24^, ^25^

HDAC inhibitors are widely investigated in cancers, which show synergistic effect with certain anticancer drugs.^26^, ^27^ HDAC inhibitors Vorinostat and Romidepsin were approved for treating refractory cutaneous T‐cell lymphoma (CTCL) clinically.^28^ Despite the promising anticancer activities of HDAC inhibitors, clinical trials with HDAC inhibitors in solid tumors have not met success. Upregulation of *multidrug resistance 1* (*MDR1*) and its encoding protein p‐gp are believed to be a typical side‐effect of HDAC inhibitors. Treatment of HDAC inhibitors, such as Apocodin, increased rhodamine‐123 efflux and modulated *MDR1* expression in Hela cells.^29^ Sodium valproate (VPA) was found to increase the expression of *MDR1* in HepG2, SW620, and KG1a cells.^30^, ^31^ Moreover, pan‐HDAC inhibitor trichostatin A (TSA) and sodium butyrate (NAB), could induce cell differentiation and accompanied with the increase of *MDR1*.^32^, ^33^ What's more, HDAC inhibitors induce the upregulation of *MDR1*, *BCRP*, *MRP7*, and *MRP8*.^34^, ^35^ Thereby, the slow progress of HDAC inhibitors in clinical research may be associated with the ATP‐binding cassette (ABC) transporters. It is urgent to find effective agents which target and inhibit HDACs without triggering the multidrug resistance.

Baicalein, a flavonoid extracted from the root of *Scutellaria baicalenesis*, has been reported to mediate growth inhibition of human leukemia cells (K562 and HL‐60).^36^, ^37^ We found that Baicalein showed inhibitory effects on HDAC‐1/8, with no effects on the expression of ABC transporter genes that can be induced by most HDAC inhibitors. Moreover, we investigated the anti‐proliferation activity of Baicalein in vivo and in vitro and its effects on differentiation and apoptosis of AML cells. In brief, we investigated the functional contribution of HDAC‐1/8 in anti‐leukemia effects, especially in CBF‐AML, and evaluated the efficacy of Baicalein in AML cells‐bearing mice model.

## 2 MATERIALS AND METHODS

### 2.1 Compounds and reagents

Baicalein (C_15_H_10_O_5_, MW: 270.24 g/mol) with the purity of 97% was determined by HPLC. For in vitro experiments, Baicalein was dissolved in dimethyl sulfoxide (DMSO, Sigma‐Aldrich, Missouri, USA) at a concentration of 0.1 M, stored at ‐20°C and diluted to a suitable concentration with RPMI‐1640 medium (Gibco, California, USA). Cells treated with the highest concentration of DMSO were used as controls in the indicated experiments.

In vivo study, Baicalein was prepared as intragastric administration (0.5% sodium carboxyl methyl cellulose) by Dr. Xue Ke from College of Pharmacy, China Pharmaceutical University. Sodium butyrate (NAB, Aladdin, Shanghai, China) and sodium valproate (VPA, Sanofi, Shanghai, China) were prepared as intraperitoneal administration (0.9% normal saline) by Dr. Xue Ke from College of Pharmacy, China Pharmaceutical University.

Fluorescein isothiocyanate (FITC) anti‐human CD14 and phycoerythrin (PE) anti‐human CD11b antibodies were obtained from eBioscience (San Diego, CA, USA). TSA (CSN12139) and PCI‐34051 (CSN16819) were obtained from CSNpharm (Chicago, USA). MG‐132 (HY‐13259) and Z‐VAD‐FMK (HY‐16658B) were obtained from MedChemExpress (Monmouth Junction, NJ, USA).

### 2.2 Cell culture

Human AML cell lines U937, THP‐1, Kasumi‐1, SKNO‐1, and ME‐1 were purchased from Cell Bank of Shanghai Institute of Biochemistry and Cell Biology. Primary leukemia cells from AML patients (The Affiliated DrumTower Hospital of Nanjing University Medical School, Nanjing, China) were collected using lymphocyte‐monocyte separation medium (Jingmei, Shanghai, China). Primary AML cells and AML cell lines were cultured in RPMI‐1640 medium, supplemented with 10% FBS, 100 U/mL of benzyl penicillin, and 100 U/mL of streptomycin in a humidified environment with 5% CO_2_ at 37°C. All cells used were passaged in our laboratory for less than 3 months after resurrection.

### 2.3 Animal models

Female NOD/SCID mice (6‐9 weeks old) (Beijing HFK Bioscience Co., Ltd, Beijing, China) were sublethally irradiated (1.8 Gy), and were engrafted with human primary AML cells (1 × 10^7^ cells per mouse) via tail vein in 24 h following the radiation treatment. Seven days later, the mice were injected by intragastric with or without Baicalein (80 mg/kg) or VPA/NAB (200 mg/kg) every other day for 4 weeks. Animals in the control group were injected with normal saline to evaluate the effects of injection on survival. Next, the animals were inspected daily for 4 weeks. Finally, peripheral blood, bone marrow (BM), and spleen cells were prepared for flow cytometry after labeling with huCD45. The BM biopsy was used to perform immunofluorescent staining.

Animals were maintained in an air‐conditioned and pathogen‐free environment (23 ± 2°C, 55 ± 5% humidity) under controlled lighting (12 h light/day) and supplied with standard laboratory food and water ad libitum throughout the experimental period. The animal study was carried out according to the regulations of the China Food and Drug Administration (CFDA) on Animal Care.

### 2.4 Target engagement analyses

For enzyme activity assay, the purified protein of HDACs were incubated with substrates that contained acetylated lysine side chains. The substrate of HDAC‐1 (#50051, BPS Bioscience, California, USA), HDAC‐2 (KDA‐21‐277, Reaction Biology Corp internally, Pennsylvania, USA), and HDAC‐3 (#50003, BPS Bioscience, California, USA) were K379‐382 (RHKK(Ac)AMC) residues of p53. The substrate of HDAC‐8 (#50008, BPS Bioscience, California, USA) was p53‐K379‐K382(RHK(Ac)K(Ac)AMC). The deacetylation of the substrates react with the lysine developer and the fluorescence were positive correlation with the level of deacetylation. Fluorescent signals were analyzed by multi‐function microplate reader. The positive control was HDAC inhibitor TSA.

For cellular thermal shift assay (CETSA) was performed to determine the direct binding between Baicalein and HDAC‐1/2/3/8 in ME‐1 cells.^38^ ME‐1 cells were pretreated with 30 µM Baicalein for 6 h. Then, collected the cells and resuspended with phosphate buffer saline (PBS) that contained protease inhibitor cocktail and transferred them into 200 µL tubes. The cells were heat shocked in the Thermal Cycler T960 (Heal Force, Hangzhou, China) at 37‐70°C for 3 min to denature proteins, then all the samples were subjected to three freeze‐thaw cycles with liquid nitrogen to lyse cells. Centrifuged the samples at 13 000 rpm for 20 min at 4°C. Added the loading buffer and prepared for western blot.

### 2.5 siRNA transfection

*HDAC‐1* and *HDAC‐8* siRNA were synthesized by GenePharma Co, Ltd (Shanghai, China). Transfection was performed using Lipofectamine 2000™ (Invitrogen, San Diego, CA) according to the manufacturer's instructions provided by the vendor. First, siRNA or the negative control and transfection reagent were diluted in serum‐free 1640, respectively. After incubated at room temperature for 20 min, the mixture was delivered into the cells. Cells were collected for further experiments after incubated for 48 h.

The siRNA sense oligonucleotides for *HDAC‐1* was 5′‐AUAAACGCAUUGCCUGUGAUCAAAGAAGAGGUCAAGUU‐3′, and the anti‐sense was 5′‐UGACCAACCAGAACACUAAGAACUCUUCUAACUUCAAA‐3′.

The siRNA sense oligonucleotides for *HDAC‐8* was 5′‐CAUCGAAGGUUAUGACUGUUGACUAUGCAGCAGCUAUA‐3′, and the anti‐sense was 5′‐CUACGUGGAUUUGGAUCUAGAUGAGAAGUACUAUCACA‐3′.

### 2.6 Differentiation assay

Cell differentiation was assessed by NBT reduction and Giemsa Staining as previously reported.^39^ Fluorescence intensity of CD11b and CD14 was analyzed with an FACS Calibur flow cytometer (Becton‐Dickinson, San Jose, CA, USA).^40^ Data were based on the examination of 10 000 cells per sample selected randomly from 5 × 10^5^ cells.

### 2.7 Western blot analysis

Western blot was performed with standard protocols.^41^ Equal amounts of protein extracts were loaded for 10% SDS‐PAGE and transferred to nitrocellulose membranes (BiTrace NT, PallCor). The membranes were blocked with 3% BSA in PBS at room temperature for 1 h, incubated with a primary antibody diluted at the indicated ratio in PBST (PBS and 0.1% Tween‐20) at 37°C for 1 h and then incubated overnight at 4°C. The membranes were washed with PBST three times and then incubated with an IRDyeTM800‐conjugated secondary antibody for 55 min at 37°C. After washing the membranes with PBST three times and then with PBS one time for 10 min each time, detection was performed with the Amersham Imager 600 System (GE, USA).

Primary antibodies against C/EBPα, HSP90, ETO, and β‐actin were obtained from Santa Cruz Biotechnology (CA, USA). Primary antibodies against HDAC‐1, Histone H3, and acetyl‐Histone H3 were products of Cell Signaling Technology (MA, USA). Primary antibodies against HDAC‐1, Histone H3, acetyl‐Histone H3, and acetyl‐p53 (K382) were purchased from Cell Signaling Technology (MA, USA). Primary antibodies against CBFβ, HDAC‐8, and pan‐acetyl‐lysine were obtained from Abclonal Technology (Wuhan, China). Alexa Fluor 488 Goat anti‐Mouse IgG (H + L) cross‐adsorbed secondary antibody was purchased from ThermoFisher Scientific (CA, USA). IRDyeTM 800‐conjugated goat anti‐mouse and goat anti‐rabbit secondary antibodies were purchased from Rockland Inc. (PA, USA).

### 2.8 Immunofluorescence

Immunofluorescence was performed as previously described.^42^ Alexa Fluor 488 donkey anti‐goat IgG (H+L) antibody was purchased from Life Technologies (CA, USA).

### 2.9 Quantitative real‐time PCR

Quantitative real‐time PCR (RT‐qPCR) was performed according to the manufacturer's instructions.^43^ The mRNA was isolated by total RNA extraction reagent (R401‐01, Vazyme, Nanjing, China). The HiScript II One Step RT‐PCR Kit (P611‐01, Vazyme, Nanjing, China) was used to reverse transcription. The cDNA was used to conduct quantitative real‐time PCR (qPCR) by using AceQ qPCR SYBR Green Master Mix (Q131‐02, Vazyme, Nanjing, China ).

The primer sequences were as follows:

*GAPDH*
Forward 5′‐GCAGGGGGGAGCCAAAAGGG‐3′
Reverse 5′‐TGCCAGCCCCAGCGTCAAAG‐3′
*DR5*
Forward 5′‐CTGTGCATTCGTCTCTCTTGG‐3′
Reverse 5′‐TGAGTCGTTTCCGTTTACCG‐3′
*PUMA*
Forward 5′‐CACCCCATCGCCTCCTTTCT‐3′
Reverse 5′‐GGAAGGGGCGCGGACTGTCG‐3′
*Noxa*
Forward 5′‐AGATGCCTGGGAAGAAG‐3′
Reverse 5′‐AGTCCCCTCATGCAAGT‐3′
*Bax*
Forward 5′‐TCAAGGCCCTGTGCACTAA‐3′
Reverse 5′‐TGAGGACTCCAGCCACAAA‐3′
*PIG5*
Forward 5′‐GAAGGATGTGGCGAAGGGA‐3′
Reverse 5′‐CCACAAGACCGTCTACCTGCA‐3′
*Bcl‐xl*
  Forward 5′‐GATGCAGGTATTGGTGAGTCGG‐3′
Reverse 5′‐ATCCACAAAAGTGTCCCAGCC‐3′
*MDR1*
Forward 5′‐CATTGGCGAGCCTGGTAG‐3′
Reverse 5′‐TCGTAGGAGTGTCCGTGGAT‐3′
*MRP8*
Forward 5′‐AAACTTCTCTGTGGGGGAGA‐3′
Reverse 5′‐GGGTGTCTGTCTCCATGTCA‐3′
*MRP7*
Forward 5′‐CATGCAAGCCACGCGGAACG‐3′
Reverse 5′‐AAGCTGGGCTGGTGGAGGGT‐3′
*BCRP*
Forward 5′‐TGCCCAGGACTCAATGCAACA‐3′
Reverse 5′‐ACAATTTCAGGTAGGCAATTGTG‐3′
*HDAC‐1*
Forward 5ʹ‐CTACTACGACGGGGATGTTGG‐3ʹ
Reverse 5ʹ‐GAGTCATGCGGATTCGGTGAG‐3ʹ
*HDAC‐8*
Forward 5ʹ‐ GGCTGCGGAACGGTTTTAAG‐3ʹ
Reverse 5ʹ‐ GCTTCAATCAAAGAATGCACCATAC‐3ʹ

### 2.10 FACS analysis of whole blood

FACS analysis of whole blood was performed as previously described.^42^ Anti‐human CD45 antibodies were obtained from Miltenyi Biotec Inc. (CA, USA).

### 2.11 Duolink assay

Duolink assay was performed by Duolink in Situ Detection Reagents Red (DUO92008) according to the manufacturer's instructions.^44^ Duolink In Situ PLA Probe Anti‐Rabbit PLUS (DUO92002) and Duolink In Situ PLA Probe Anti‐Mouse MINUS (DUO92004) were purchased from Sigma‐Aldrich.

### 2.12 Immunoprecipitation

Cells were pretreated with or without Baicalein (30 µM). Cell lysate was incubated with appropriate concentration of antibody and 20 µL protein A/G‐conjugated beads (Santa Cruz) at 4°C overnight. After washing three times with RIPA buffer (Thermo Fisher), samples were collected and re‐suspended in 20 µL SDS‐sample buffer (0.5 M Tris‐HCl, pH 6.8, 20% glycerol, 2% SDS, 5% 2‐mercaptoethanol and 4‰ bromophenol blue) and boiled for 10 min. Then the samples were subjected to western blot.

### 2.13 Statistical analysis

All data were expressed as mean ± SD. The data shown were obtained from triplicate independent parallel experiments. Statistical analysis of multiple group comparisons was performed by one‐way analysis of variance (ANOVA) followed by the Bonferroni post hoc test. Comparisons between two groups were analyzed using two‐tailed Student's *t*‐tests. A *P*‐value < .05 was considered statistically significant.

## 3 RESULTS

### 3.1 Baicalein inhibited HDAC‐1/8 activity without affecting the expression of HDAC inhibitors‐associated ABC transporter genes

**FIGURE 1:**
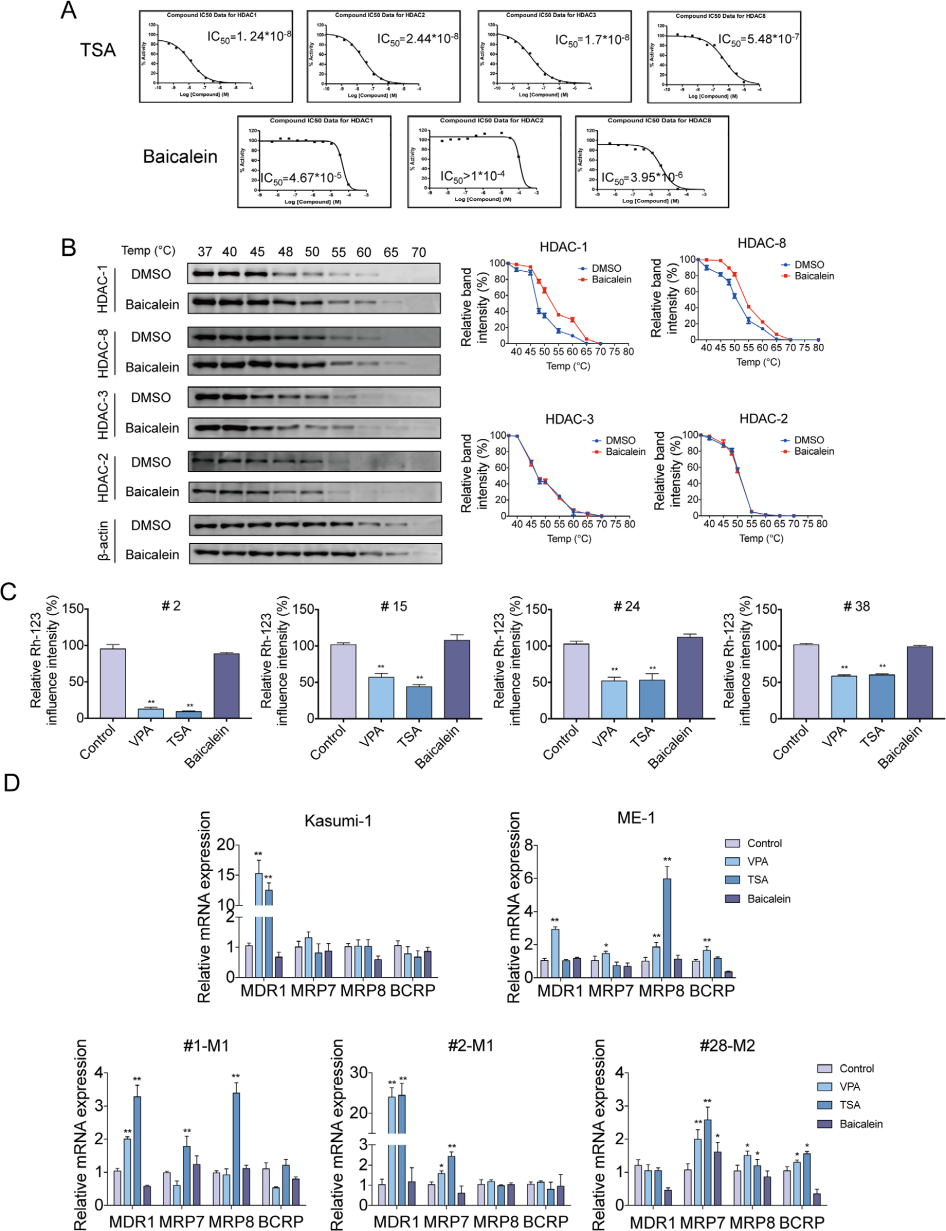
Effects of Baicalein on HDAC‐1/8 activity and the expression of HDAC inhibitors‐associated ABC transporter genes. A, HDACs enzyme activity assay of Baicalein and TSA. B, ME‐1 cells were treated with Baicalein (30 µM) for 6 h. The temperatures were between 37 and 70°C and used to perform ITDR_CETSA_ assay directed toward HDAC‐1, HDAC‐2, HDAC‐3, and HDAC‐8. Data were first normalized by setting the highest and lowest value in each set to 100% and 0%, respectively. Data were obtained in the presence of the Baicalein (red square) as positive control and DMSO (blue circle) as negative control. C, Primary AML cells (#2, #15, #24, #38) were treated with 165 nM TSA or 3 mM VPA for 24 h or 30 µM Baicalein for 96 h. The accumulation of Rh‐123 were detected by flow cytometry. D, Kasumi‐1, ME‐1 cells, and primary AML cells (#1, #2, #28) were treated with 165 nM TSA or 3 mM VPA for 24 h or 30 µM Baicalein for 96 h. Effects of HDAC inhibitors on *MDR1*, *MRP7*, *MRP8*, and *BCRP* expression were analyzed by RT‐qPCR. The data represent the mean ± SD of three different experiments. Asterisks denote statistically significant *P < .05; **P < .01; Differences compared with controls by one‐way ANOVA

Emerging data now implicate histone modification is considered for a new therapeutic strategy in cancer including AML.^45^ Histone acetylation is mediated by histone acetyltransferases, while acetyl groups are removed by HDACs.^46^ To investigate the effect of Baicalein on HDACs activity, we performed enzyme activity assay. Results showed that Baicalein inhibited the enzyme activity of HDAC‐1 and HDAC‐8 in a concentration‐dependent manner but not HDAC‐2 and HDAC‐3. And the IC_50_ values of Baicalein on HDAC‐1 and HDAC‐8 were 4.67 × 10^−5^ and 3.95 × 10^−6^ M, respectively (Figure 1A). Meanwhile, TSA was selected as a reference drug. We found that TSA inhibited the enzyme activity of HDAC‐1, HDAC‐2, HDAC‐3, and HDAC‐8, the IC_50_ values were showed in Figure 1A. What's more, Cellular thermal shift assay (CETSA) was used to study thermal stabilization of proteins upon ligand binding. Results showed that the apparent aggregation temperature (T_agg_) values for HDAC‐1, HDAC‐2, HDAC‐3, and HDAC‐8 of DMSO group were 47.78, 50.69, 46.01, and 50.98°C while the T_agg_ values for HDAC‐1, HDAC‐2, HDAC‐3, and HDAC‐8 of Baicalein‐treated group were 53.23, 50.49, 45.84, and 54.13°C, respectively. We applied this method to further confirm the interaction between Baicalein and HDAC‐1 and HDAC‐8 but not HDAC‐2 and HDAC‐3 (Figure 1B).

Multiple studies indicated that the exposure of cancer cells to HDAC inhibitors could induce the activation of several drug resistance‐associated ABC transporters, which in turn lead to a broad‐spectrum of drug resistance effect in the treatment of cancer.^34^ Therefore, in order to further investigate the effect of Baicalein on the drug resistance, we used Rhodamine‐123 (Rh‐123) to evaluate the distribution of drugs on primary AML cells.^47^ Compared with HDAC inhibitors VPA (3 mM) and TSA (165 nM), Baicalein showed little influence on the transport of Rhodamine123 (Figure 1C; Table 1), revealing that traditional HDAC inhibitors decreased the intracellular accumulation of Rh‐123 while Baicalein hardly decreased its accumulation. *MDR1* is an ATP‐binding cassette ABCB1 and a prognostic factor of AML treatment failure.^48^ Moreover, multidrug resistance of HDAC inhibitors is associated with *BCRP*, *MPR7*, and *MRP8*.^49^, ^50^, ^51^ The mRNA levels of ABC transporter genes *MDR1*, *BCRP*, *MRP7*, and *MRP8* were dramatically increased after exposure to either VPA (3 mM) or TSA (165 nM) for 24 h in AML cell lines and relapsed AML patient samples (#1, #2, and #28). However, the mRNA levels of those genes changed slightly after treatment of Baicalein for 96 h (Figure 1D).

**TABLE 1:** Clinical data for patient samples with AML

Patient NO.	Source	FAB	PBblast %	BMblast %	WBC	Cytogenetics	Status
#1	PB	M1	87.2	94		CEBPA/TET2	Relapsed
#2	PB	M1	98	98	369.3	OD	Relapsed
#3	PB	M1				OD	New
#4	PB	M1		95	190.02	DNTM3A/FLT3‐ITD	New
#5	PB	M1		98	128.3	NPM1/WT1	New
#6	PB	M1		93	94.8	OD	New
#7	PB	M1	90.8	92	102.52	OD	New
#8	PB	M2b	55	80.96	8.5	AML1‐ETO/WT1	New
#9	PB	M2b	2.79	35.5		AML1‐ETO/WT1	New
#10	PB	M2b		7	7.2	CEBPA/AML1‐ETO	New
#11	PB	M2b			32.5	AML1‐ETO	New
#12	PB	M2b			89.07	TET2/WT1/AML1‐ETO	New
#13	PB	M2b	44	<20	1.8	AML1‐ETO	New
#14	PB	M2b		19	15.7	AML1‐ETO	New
#15	PB	M2b			8.3	AML1‐ETO/c‐kit	Relapsed
#16	PB	M2a	94		111.4	OD	New
#17	PB	M2a	68	64	180.3	FLT3‐ITD	New
#18	PB	M2a	21	82		DNTM3A/CEBPA	New
#19	PB	M2a		69	19.8	WT1/HOX11	Relapsed
#20	PB	M2a		94.83	43.84	OD	New
#21	PB	M2a	57.5	28	14.1	WT1/EVI1/DNTM3A	New
#22	PB	M2a		42.5	4.9	CEBPA	New
#23	PB	M2a			30.3	AML1‐ETO	New
#24	PB	M2a	95	86.5	295.9	OD	New
#25	PB	M2a	29	49.26	2.5	WT1/CEBPA	New
#26	PB	M2a	25.5	30.88	1.8	OD	New
#27	PB	M2a		97	72.3	OD	New
#28	PB	M2	92	90	242.24	PHF6/ASXL1/PH6	Relapsed
#29	PB	M2			192.96	AML1‐ETO	New
#30	PB	M2	23	19.68	2.67	WT1/HOX11	New
#31	PB	M2	22.8	46	28.8	TET2/CEBPA	New
#32	PB	M2	11		10.8	OD	New
#33	PB	M2				OD	New
#34	PB	M2			13.7	OD	New
#35	PB	M4				OD	New
#36	PB	M4b	2	20.5	300.07	OD	New
#37	PB	M4b	81	20	170	FLT3‐ITD/NPM1	Relapsed
#38	PB	M5			21.41	WNT1	Relapsed
#39	PB	M5		71		TET2/IDH2/NPM1/FLT3	New
#40	PB	M5b	50		24.7	CBFB‐MYH11/WT1	New
#41	PB	M5b	12	27.5	2.9	WT1/TLS‐ERG	New
#42	PB	M4	94	94.4		CBFB‐MYH11	New
#43	PB	M4	39	43.2		OD	New

Abbreviations: BM, bone marrow; FAB, French–American–British; OD, outside diagnosis; PB, peripheral blood; WBC, white blood cells count.

### 3.2 Baicalein inhibited cell growth and induced differentiation of AML cell lines and primary AML cells

**FIGURE 2:**
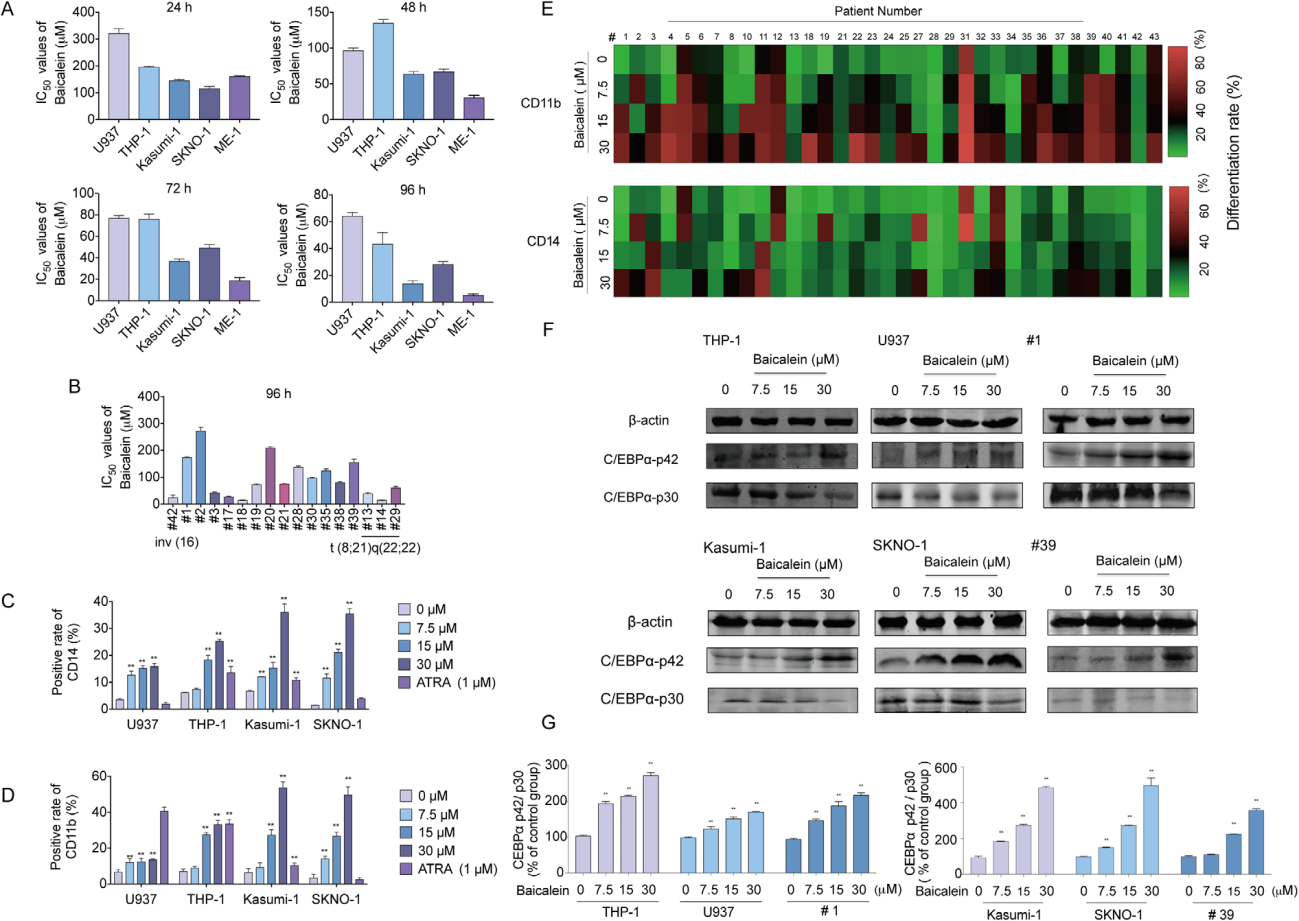
Effects of Baicalein on the growth and differentiation of AML cell lines and primary AML cells. A, IC_50_ values of Baicalein in U937, THP‐1, Kasumi‐1, SKNO‐1, and ME‐1 cells after treatment for 24, 48, 72, and 96 h. B, IC_50_ values of Baicalein in primary AML cells after treatment for 96 h. C,D, U937, THP‐1, Kasumi‐1, and SKNO‐1 cells were treated with Baicalein (0, 7.5, 15, and 30 µM) and ATRA (1 µM) for 96 h. The expression of CD11b/CD14 was measured by flow cytometry. E, Hot maps of the expression of CD11b/CD14 after Baicalein (0, 7.5, 15, and 30 µM) treatment for 96 h in primary AML cells. The percentages of cells expressing CD11b and CD14 were detected by flow cytometry. F,G, Expression levels of C/EBPα‐p42 and C/EBPα‐p30 were analyzed by western blot after treatment with Baicalein (0, 7.5, 15, and 30 µM) for 96 h in U937, THP‐1, Kasumi‐1, SKNO‐1, and primary AML cells (#1, #39). β‐Actin was used as a loading control. The data represent the mean ± SD of three different experiments. Asterisks denote statistically significant *P < .05; **P < .01; Differences compared with controls by one‐way ANOVA

AML cell lines including U937 and THP‐1, CBF‐AML cells including Kasumi‐1 with t(8;21), SKNO‐1 with t(8;21), and ME‐1 with inv(16), and primary AML cells were cultured in the absence or presence of Baicalein (0‐256 µM). Results showed that the treatment of Baicalein induced significant inhibition of cell viability in those cells, and have shown its superior performance in CBF‐AML by CCK8 assay (Figure 2A,B; Figure S1A‐C).

We then examined the effect of Baicalein on the differentiation progression in AML cells. To confirm the differentiation induction effect of Baicalein on AML cells, we examined the expression of CD11b, a marker of myeloid differentiation, and CD14, a marker of monocytic maturation by FACs. The percentage of CD11b and CD14 positive were both increased after Baicalein treatment for 96 h in U937, THP‐1, Kasumi‐1, SKNO‐1 cells, and primary AML cells (Table 2; Figure 2C‐E). Notably, Baicalein showed better differentiation induction potential in t(8;21)q(22;22) AML cells. However, Baicalein has no influence on the differentiation of ME‐1 cells (data not shown). Furthermore, the NBT‐reduction activity of U937, THP‐1, Kasumi‐1, SKNO‐1 cells, and primary AML cells (#14, #41) also dramatically increased after treatment with Baicalein for 96 h (Figure S1D). The expression of C/EBPα‐p42 that was vital in the development of granulocyte and neutrophil development acting as a tumor suppressor in the hematopoietic system was upregulated by Baicalein in AML cells. Interestingly, the expression of C/EBPα‐p30 was downregulated in cell lines including THP‐1, U937, and primary AML cells (#1, #39), however, it mildly decreased in t(8;21) AML, such as Kasumi‐1 and SKNO‐1 (Figure 2F,G). These results suggested that Baicalein inhibits cell growth and induced differentiation accompanied with an increase of C/EBPα‐p42/ C/EBPα‐p30 ratio in AML cell lines and primary AML cells.

**TABLE 2:** Expression of CD11b/CD14 in primary AML cells

	CD11b positive cells (%)				CD14 positive cells (%)			
	Baicalein (µM)				Baicalein (µM)			
Patient No.	0	7.5	15	30	0	7.5	15	30
#1	4.38	18.33	51.9	61.16	1.78	3.99	12.12	42.36
#2	16.1	48	13	31	16.2	47	14	31
#3	11.48	20.8	40.9	52.11	15.09	23.5	43.6	53.93
#4	0.41	5.84	66.13	76.16	0.48	4.8	19.66	13.44
#5	38.61	60.77	64.99	66.1	40.05	45.08	23.32	13.39
#6	26.56	38.09	53.12	45.98	12.79	20.44	21.21	32.61
#7	25.78	27.77	26.94	31.02	20.55	21.92	11.26	12.19
#8	8.14	6.41	16.57	55.87	3.88	2.62	5.04	29.5
#10	1.97	6.81	59.38	30.37	1.97	19.87	17.66	37.6
#11	28.57	58.12	59.62	63.43	5.14	9.9	51.43	58.6
#12	37.94	48.21	55.62	56.64	39.1	52.27	11.26	15.68
#13	4.6	7.47	16.61	21.31	1.1	3.72	6.98	7.94
#18	10.71	17.06	30.92	75.56	6.47	9.17	13.92	16.18
#19	1.5	33.83	36.28	43.23	2.87	46.75	21.32	19.96
#21	20.3	20.34	20.61	19.37	11.29	10.53	14.12	12.72
#22	10.7	24.07	39.91	80.02	7.92	13.74	24.14	25.67
#23	18.12	29.56	35.26	61.07	14.28	25.22	14.93	18.7
#24	8.46	25.73	22.71	20.67	6.97	13.67	13.09	14.6
#25	9.23	17.98	36.21	50.23	7.94	14.33	18.99	25.44
#27	9.17	9.36	11.4	44.48	6.14	60.6	6.53	38.48
#28	5.32	2.08	4.35	5.96	3.17	1.28	2.34	3.96
#29	21.2	28.28	19.44	36.27	6.49	7.03	7.64	13.67
#31	69.19	85.22	89.04	92.8	59.74	77.34	13.89	11.29
#32	16.52	25.9	34.52	50.67	2.47	8.45	17.85	34.23
#33	11.02	9.7	29.5	44.78	45.3	49.14	36.92	32.85
#34	18.03	3.61	5.47	18.2	1.37	0.8	5.01	10.84
#35	16.8	45.04	38.24	38.02	13.53	21.69	19.65	17.43
#36	28.75	31.82	52.37	65.63	22.29	25.21	10.37	13.96
#37	6.11	14.95	30.19	49.45	2.62	11.44	18.69	27.76
#38	27.32	26.16	33.66	39.45	24.71	24.09	29.46	34.49
#39	25.3	60.38	64.87	66.44	9.9	19.02	24.34	35.52
#40	6.53	55.32	54.38	61.34	9.42	18.2	20.1	23.2
#41	26.42	28.9	34.8	38.33	7.13	14.98	23.32	30.66
#42	9.2	13.2	12.3	11.4	4.5	2.5	4.09	4.05
#43	33.76	35.4	35.8	44.79	14.13	14.21	14.29	22.19

### 3.3 Baicalein promoted proteasome‐dependent degradation of HDAC‐1 in AML cells

**FIGURE 3:**
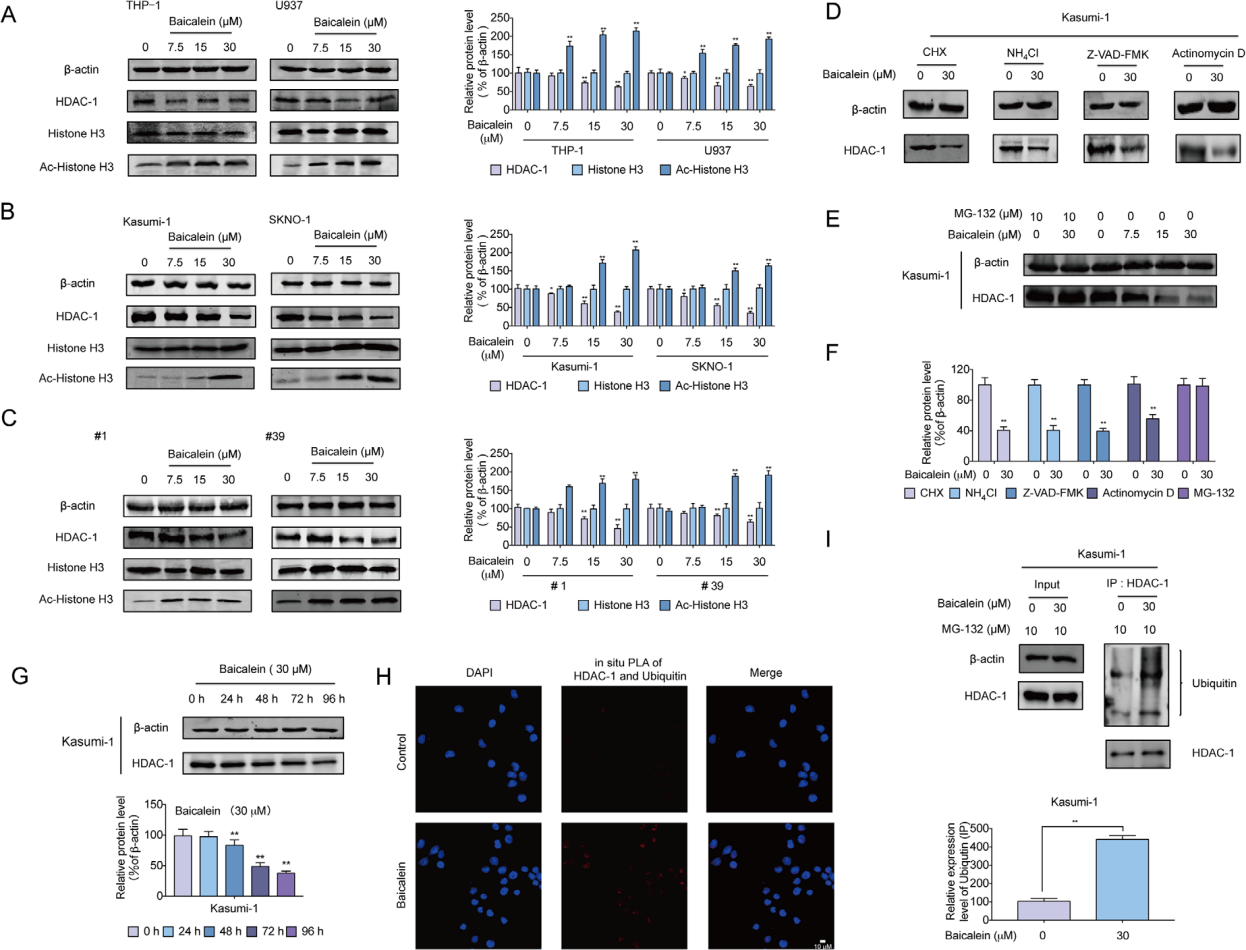
Baicalein promoted the degradation of HDAC‐1 via proteasome‐dependent pathway. A‐C, Expression levels of HDAC‐1, Histone‐H3, and acetylated‐Histone H3 (AC‐ Histone H3) were analyzed by western blot after treatment with Baicalein (0, 7.5, 15, and 30 µM) for 96 h in U937, THP‐1, Kasumi‐1, SKNO‐1 cells, and primary AML cells (#1, #39). β‐Actin was used as a loading control. D‐F, Kasumi‐1 cells were treated with 15 µg/mL CHX or 5 mM NH_4_Cl or 20 µM z‐VAD‐FMK or 5 µg/mL Actinomycin D or 10 µM MG‐132, and/or 30 µM Baicalein for 96 h. Western blot was performed to detect the expression of HDAC‐1. β‐Actin was used as a loading control. G, Kasumi‐1 cells were treated with 30 µM Baicalein for 0, 24, 48, 72, and 96 h. Expression levels of HDAC‐1 were analyzed by western blot. β‐Actin was used as a loading control. H, Kasumi‐1 cells were co‐treated with 10 µM MG‐132 and/or 30 µM Baicalein for 48 h. Representative images of Duolink in situ PLA using rabbit anti‐Ubiquitin, mouse anti‐HDAC‐1 antibodies, and PLA probes. Red foci indicate HDAC‐1‐Ubiquitin interactions, DAPI‐stained nuclei are in blue. They were detected by a confocal laser scanning microscope (FluoView FV1000, Olympus, Tokyo, Japan). I, Kasumi‐1 cells were co‐treated with 10 µM MG‐132 and/or 30 µM Baicalein for 48 h. Then cells were immunoprecipitated with anti‐HDAC‐1 antibody, followed by western blot analysis. The data represent the mean ± SD of three different experiments. Asterisks denote statistically significant *P < .05; **P < .01; Differences compared with controls by one‐way ANOVA

Pharmacologic inhibition of HDACs induced differentiation, proliferation inhibition, and apoptosis of AML cells.^52^ We found that Baicalein mildly inhibited HDAC‐1 activity, while obviously inhibited HDAC‐1 expression in U937, THP‐1, Kasumi‐1, SKNO‐1 cells, and primary AML cells (#1, #39) (Figure 3A‐C). Meanwhile, the acetylation of Histone H3 was upregulated by Baicalein in those cells (Figure 3A‐C). Notably, the high background expression of HDAC‐1 was consistent with the potent growth inhibition and differentiation induction by Baicalein in AML cells and primary AML cells (Figure 2C‐E; Figure S1A; Table 2). Even primary leukemia AML cells (#2, #28) with low background expression of HDAC‐1, Baicalein inhibited the expression of HDAC‐1 protein as well (Figure S2B).

To understand the mechanism of Baicalein‐induced HDAC‐1 reduction, we first determined whether Baicalein affected the expression of HDAC‐1 at transcriptional level. Kasumi‐1 was treated with cycloheximide (CHX), a protein synthesis inhibitor and results showed that CHX did not affect Baicalein‐induced HDAC‐1 decrease (Figure 3D,F). We further adopted Actinomycin D (5 µg/mL), a transcription inhibitor, and results suggested that the downregulation of HDAC‐1 was not due to transcription inhibition (Figure 3D,F). Moreover, either NH_4_Cl, a lysosome inhibitor, or z‐VAD‐FMK, a broad‐spectrum caspase inhibitor, was respectively applied in co‐treatment with Baicalein. Results showed that Baicalein‐triggered degradation of HDAC‐1 had nothing to do with lysosome‐ and caspase‐dependent pathways (Figure 3D,F). Finally, we found that the Baicalein‐induced degradation of HDAC‐1 was blocked by MG‐132, a proteasome inhibitor, indicating that the degradation of HDAC‐1 by Baicalein may be dependent on ubiquitin proteasome pathway (Figure 3D‐F). Results of western blot showed that HDAC‐1 began to degrade after treated by Baicalein for 48 h (Figure 3G). Furthermore, we found that Baicalein increased the conjugation of HDAC‐1 and ubiquitin in AML cells, which were confirmed by immunoprecipitation (IP) and Duolink assay (Figure 3H,I). Thus, the degradation of HDAC‐1 by Baicalein may be involved in the ubiquitin‐proteasome.

### 3.4 Baicalein induced the degradation of AML1‐ETO via decreasing its interaction with HSP90

**FIGURE 4:**
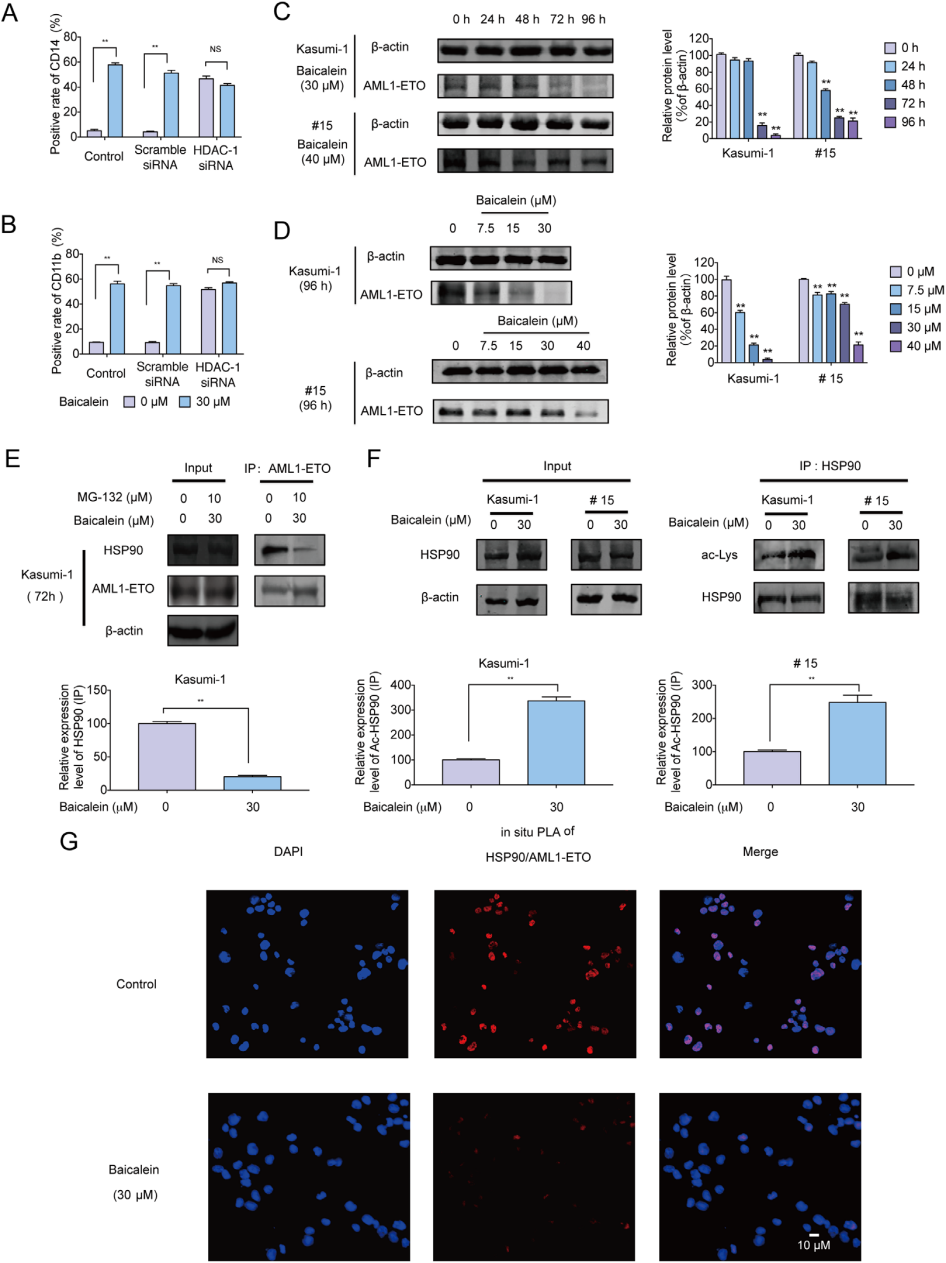
Baicalein interrupted the stability of AML1‐ETO‐HSP90 complex and induced differentiation of AML cells via HDAC‐1. A,B, The expression of HDAC‐1 in Kasumi‐1 cells was knocked down by *HDAC‐1* siRNA, and then the cells were treated with 30 µM Baicalein for 96 h. The expression of CD11b/CD14 was next detected by flow cytometry. C, Kasumi‐1 cells were treated with 30 µM Baicalein for 0, 24, 48, 72 and 96 h. Primary AML cells (#15) were treated with 40 µM Baicalein for 0, 24, 48, 72, and 96 h. Expression levels of AML1‐ETO were analyzed by western blot. β‐Actin was used as loading controls. D, Kasumi‐1 cells were treated with Baicalein (0, 7.5, 15, and 30 µM) for 96 h. Primary AML cells (#15) were treated with Baicalein (0, 7.5, 15, 30, and 40 µM) for 96 h. Expression levels of AML1‐ETO were analyzed by western blot. β‐Actin were used as loading controls. E, Kasumi‐1 cells were co‐treated with 10 µM MG‐132 and/or 30 µM Baicalein for 72 h. IP assay of HSP90 and AML1‐ETO was analyzed. Western blot was performed with the indicated antibodies. β‐Actin was used as a loading control. F, Kasumi‐1 cells were treated with 30 µM Baicalein for 96 h. IP assay of HSP90 and pan‐acetalyed‐lysine (Ac‐Lys) in Kasumi‐1 cells and primary AML cells (#15) were performed. Western blot were performed with the indicated antibodies. β‐Actin was used as loading controls. G, Kasumi‐1 cells were co‐treated with 10 µM MG‐132 and/or 30 µM Baicalein for 72 h. Representative images of Duolink in situ PLA using rabbit anti‐HSP90, mouse anti‐ETO antibodies, and PLA probes. Red foci indicate HSP90 and AML1‐ETO interaction, DAPI‐stained nuclei are in blue. They were detected by a confocal laser scanning microscope (FluoView FV1000, Olympus, Tokyo, Japan).The data represent the mean ± SD of three different experiments. Asterisks denote statistically significant *P < .05; **P < .01; Differences compared with controls by one‐way ANOVA

To investigate whether the differentiation induction of AML cells by Baicalein was dependent on HDAC‐1 expression, Kasumi‐1 cells were transfected with *HDAC‐1* small interfering RNA (siRNA). The efficacy of transfection was monitored by RT‐qPCR (Figure S3A). Cell differentiation analyses were subsequently performed by using FACS assay. Notably, *HDAC‐1* knockdown showed a certain degree of cell differentiation, but there was no significant enhance of in the degree of differentiation after treatment of Baicalein, suggesting that the induction of differentiation of AML cells by Baicalein may depend on the inhibitory effect of HDAC‐1 (Figure 4A,B).

The molecular chaperone protein HSP90 stabilized client oncogenic proteins like AML1‐ETO that recruited transcription repression complex including HDAC‐1 to repress AML1‐regulated genes.^53^ Baicalein inhibited the expression of AML1‐ETO in Kasumi‐1 and primary AML cells (#15) with t(8;21) in time‐ and dose‐dependent manner (Figure 4C,D). We further adopted Actinomycin D (5 µg/mL) and CHX (15 µg/mL), and results suggested that the change of AML‐ETO protein was not affected by transcription and protein synthesis (Figure S3B,C). Further results showed that the protein interaction between AML1‐ETO and HSP90 was interrupted by Baicalein, which might be caused by acetylation on lysine residues of HSP90 (Figure 4E‐G). Taken together, our results suggested that Baicalein induced the degradation of AML1‐ETO via increasing HSP90 acetylation so as to interrupt their interaction.

### 3.5 Baicalein restored p53 acetylation and diminished survival of CD34^+^ cells in inv(16) AML cells

**FIGURE 5:**
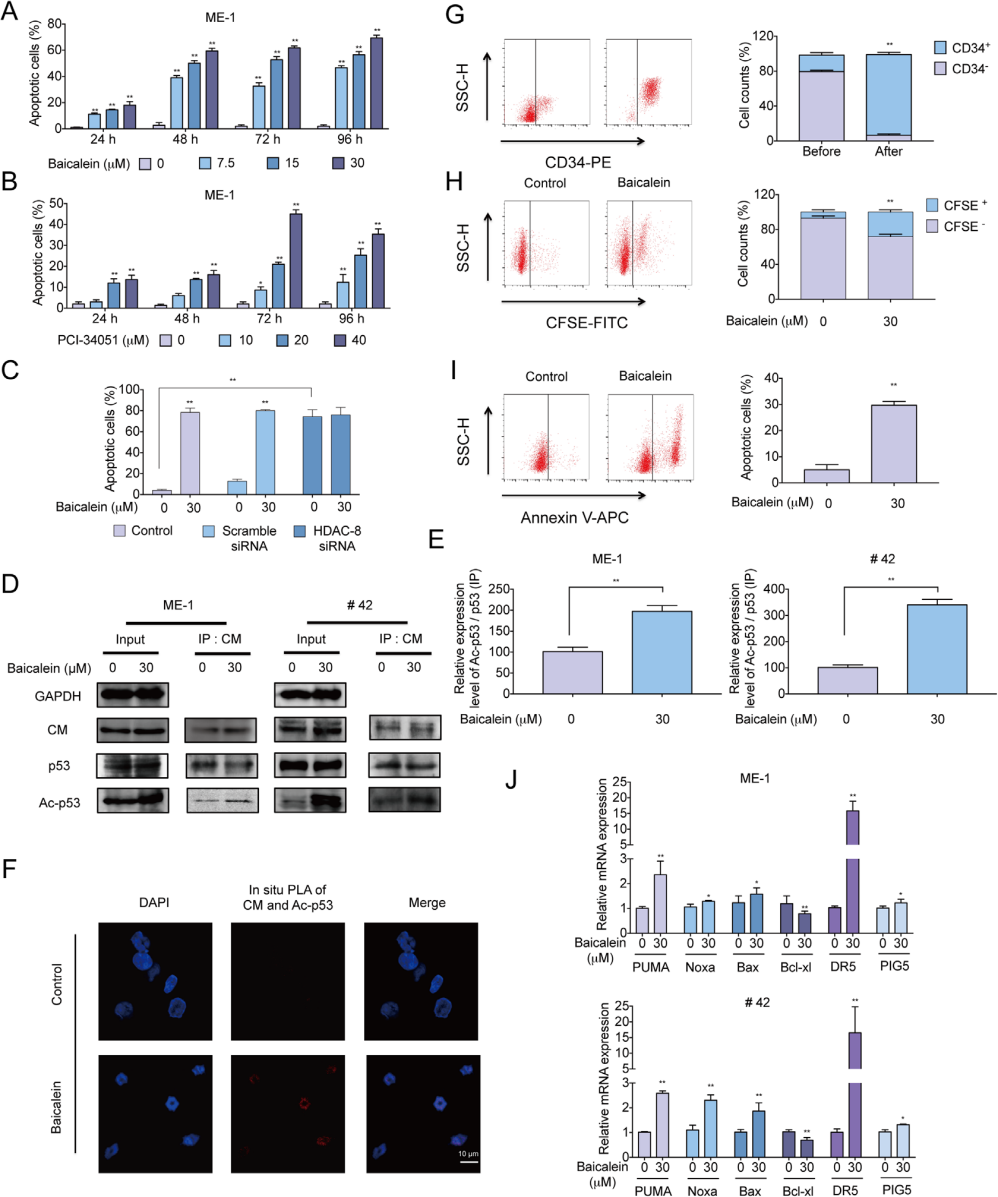
Effect of Baicalein on CM‐p53‐HDAC‐8 complex and survival of CD34^+^ cells in inv(16) AML. A,B, ME‐1 cells were treated with Baicalein (0, 7.5, 15, and 30 µM) or PCI‐34051 (0, 10, 20, and 40 µM) for 24, 48, 72, and 96 h. The apoptosis were measured using Annexin V/PI double‐staining assay by flow cytometry. C, The expression of HDAC‐8 in ME‐1 cells was knocked down by *HDAC‐8* siRNA, and then the cells were treated with 30 µM Baicalein for 48 h. The apoptosis were measured using Annexin V/PI double‐staining assay by flow cytometry. D,E, ME‐1 and primary AML cells (#42) were treated with 30 µM Baicalein for 48 h. IP assay of CBF‐β and p53 or acetalyed‐p53 (AC‐p53) were performed. Western blot was performed with the indicated antibodies. GAPDH were used as loading controls. F, Representative images of Duolink in situ PLA using rabbit anti‐CBF‐β, mouse anti‐Ac‐p53 antibodies and PLA probes were shown after Baicalein (30 µM) treatment for 48 h in ME‐1 cells. Red foci indicate CM and Ac‐p53 interaction, DAPI‐stained nuclei are in blue. They were detected by a confocal laser scanning microscope (FluoView FV1000, Olympus, Tokyo, Japan). G, CD34^+^ cells were isolated from primary AML cells (#42) by MACS. CD34^+^ cells were gathered after magnetic separation. H, CFSE staining assay of the purified CD34^+^ cells from primary AML cells (#42). The CD34^+^ cells were treated with Baicalein (30 µM) for 48 h. The expression of CFSE was performed by flow cytometry. I, Annexin V staining assay of the purified CD34^+^ cells from primary AML cells (#42). The CD34^+^ cells were treated with Baicalein (30 µM) for 48 h. The expression of Annexin V was performed by flow cytometry. J, Effects of Baicalein on the expression of downstream genes of p53 in ME‐1 cells and primary AML cells (#42). Those cells were treated with 30 µM Baicalein for 48 h, and then the genes expression was analyzed by RT‐qPCR. The data represent the mean ± SD of three different experiments. Asterisks denote statistically significant *P < .05; **P < .01; Differences compared with controls by one‐way ANOVA

We have so far confirmed the effect of Baicalein on HDAC‐8 inhibition, and have shown its superior performance in inv(16) AML by CCK8 assay (Figures 1A,B and 2A). Further data showed that Baicalein dramatically induced apoptosis in ME‐1 cells and primary inv(16) AML cells (#42) (Figure 5A; Figure S4A). Meanwhile, we used HDAC‐8 inhibitor PCI‐34051 to detect apoptotic induction in ME‐1 cells. Results showed that the apoptosis could be induced by PCI‐34051, but the efficacy was not as good as Baicalein (Figure 5B). We speculated HDAC‐8 inhibitor could induce expression of resistance‐related genes, we next detected the expression of ABC transporter genes induced by PCI‐34051 and Baicalein in ME‐1 cells. Results showed that PCI‐34051 significantly upregulated mRNA expression of *MDR1*, *BCRP*, *MPR7*, and *MRP8* compared to Baicalein (Figure S4B). We also knocked down the expression of *HDAC‐8* to verify the pharmacodynamic mechanism of Baicalein on inv(16) AML cells. The efficacy of transfection monitored using RT‐qPCR (Figure S4C). Results showed that knockdown of *HDAC‐8* in ME‐1 cells could induce apoptosis to a certain degree. However, after transfected with *HDAC‐8* siRNA, Baicalein does not enhance apoptotic effects (Figure 5C).

Recently research showed that CM fusion protein bind to p53 and HDAC‐8 to mediate CM‐induced deacetylation of p53 in inv(16) AML.^54^ IP assay and Duolink assay were then performed, which showed that Baicalein increased the acetylation of CM‐bound p53 without affecting p53 expression (Figure 5D‐F). The mRNA level of p53 downstream genes were also dramatically increased by Baicalein in ME‐1 and primary AML cells (#42) (Figure 5J). Inhibition of HDAC‐8 selectively induced apoptosis of human inv(16) AML stem and progenitor cells.^54^ We isolated CD34^+^ hematopoietic progenitor cells from #42 cells by magnetic activated cell sorting (MACS) (Figure 5G). Notably, Baicalein increased the apoptosis proportion in CD34^+^ cells that was modulated by CM‐p53‐HDAC‐8 complex (Figure 5H,I). Overall, the results suggest that Baicalein modulated the CM‐p53‐HDAC‐8 complex via inhibition of HDAC‐8, resulting in an ascendant performance of Baicalein on inv(16) AML.

### 3.6 Baicalein showed anti‐leukemic activity in AML cells‐bearing NOD/SCID mice

**FIGURE 6:**
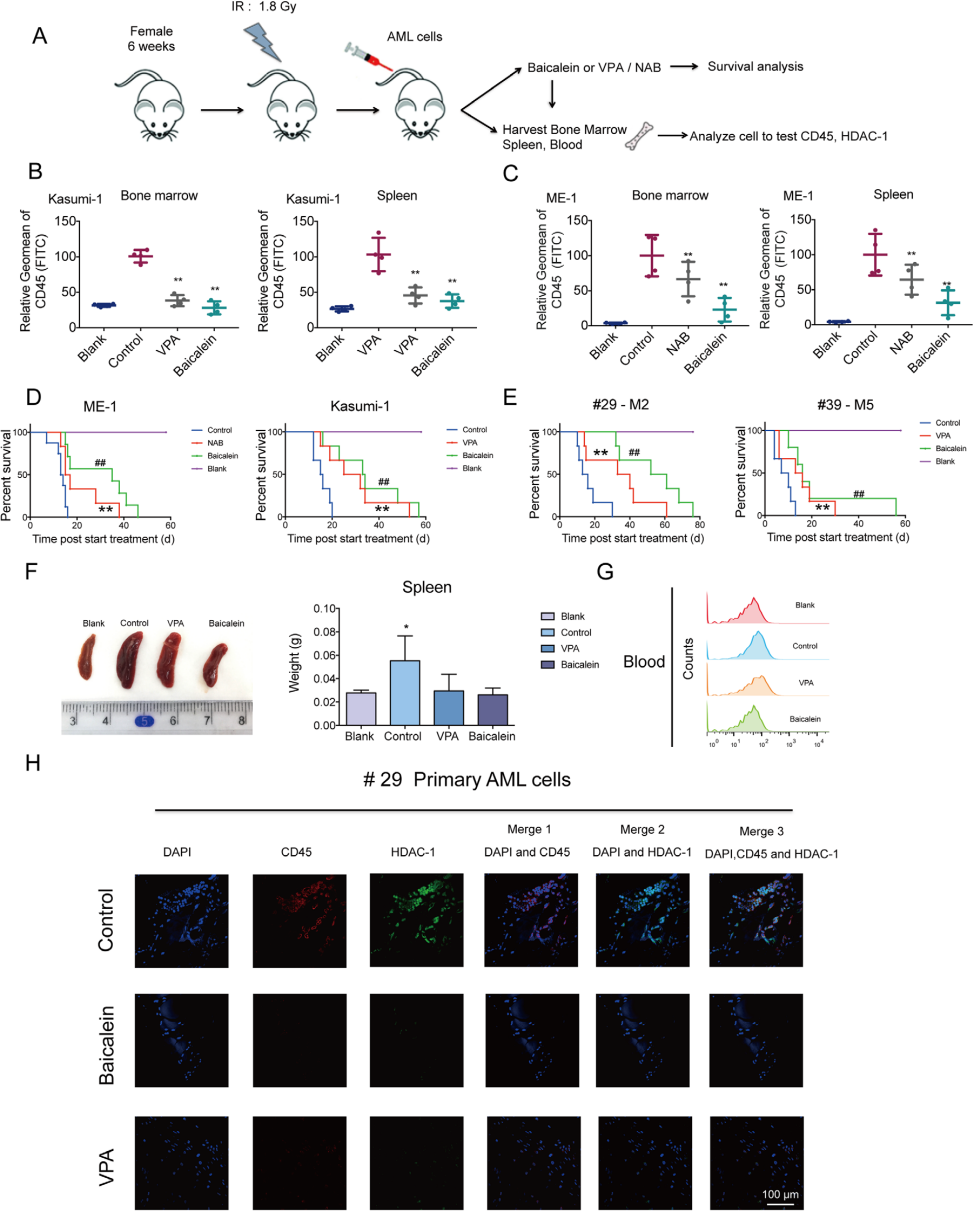
Anti‐leukemic activity of Baicalein in AML cells‐bearing NOD/SCID mice. A, Flowchart of evaluation the effect of Baicalein on AML cells‐bearing NOD/SCID mice. B,C, huCD45 expression were examined in cells come from bone marrow and spleen of each group of Kasumi‐1 and ME‐1 cells‐bearing mice by flow cytometry. D, Kaplan‐Meier survival plots for ME‐1, Kasumi‐1 cells‐bearing NOD/SCID mice were shown. E, Kaplan‐Meier survival plots for primary AML cells (#29, #39)‐bearing NOD/SCID mice were shown. F, Effects of Baicalein (80 mg/kg) and VPA (200 mg/kg) on the weight of Spleen in primary AML cells (#29)‐bearing NOD/SCID mice. And typical photos of spleen were shown. G, huCD45 expression were examined in cells come from the blood samples from four mice (#29) of each group by flow cytometry analyses. H, BM samples from three mice (#29) of each group were collected and sections were performed. Immunofluorescence and costained with huCD45‐PE (red fluorescence) and anti‐HDAC‐1 (primary)/Alexa Fluor 488 donkey anti‐goat (secondary) antibody combinations (green fluorescence), as well as DAPI (blue fluorescence). They were detected by confocal microscopy (FV1000; Olympus) with FV10‐ASW2.1 acquisition software (Olympus) at room temperature. The data represent the mean ± SD of three different experiments. Asterisks denote statistically significant *P < .05; **P < .01; Differences compared with controls by one‐way ANOVA

To further investigate the anti‐leukemia activity of Baicalein in vivo, Kasumi‐1 and ME‐1 cells were injected in NOD/SCID mice via tail vein (Figure 6A). After engrafted for 7 days, the mice were randomly assigned. We selected HDAC inhibitor VPA (200 mg/kg) in t(8; 21)q(22;22) AML cells‐bearing mice. Meanwhile, another pan‐HDAC inhibitor NAB (200 mg/kg) was chosen as a positive control in inv(16) AML cells‐bearing mice group because of its dominant position on the inhibition of HDAC‐8. We found that Baicalein significantly reduced the amount of huCD45^+^ cells, a marker of human leukocyte, in spleen and bone marrow of Kasumi‐1 and ME‐1 cells‐bearing mice, while either VPA or NAB showed mildly decrease of the huCD45^+^ cells (Figure 6B,C). Further results showed that administration of either Baicalein or VPA/NAB dramatically prolonged survival compared to control group while Baicalein showed a better survival prolonging capacity (Figure 6D, Table 3).

**TABLE 3:** Median survival of AML cells‐bearing NOD/SCID mice

Kasumi‐1 cells‐bearing NOD‐SCID mice		ME‐1 cells‐bearing NOD‐SCID mice	
	Median survival (Days)		Median survival (Days)
Control	15.5	Control	13.5
VPA	28.5	NAB	16
Baicalein	33.5	Baicalein	35

#29 sample‐bearing NOD‐SCID mice		#39 sample‐bearing NOD‐SCID mice	
	Median Survival (Days)		Median Survival (Days)
Control	14.5	Control	8.5
VPA	36.5	VPA	14.5
Baicalein	56.5	Baicalein	16

To assess the in vivo anti‐leukemia effect of Baicalein in human primary AML cells, we established two primary AML cells‐bearing mice models engrafted by primary AML cells (#29) with t(8;21)q(22;22) and #39, a non‐CBF‐AML sample respectively. After administration of Baicalein (80 mg/kg) and VPA (200 mg/kg) every 2 days for 4 weeks, the survival was recorded. Baicalein showed a superior status of the median survival duration in primary AML cells (#29) cells‐bearing mice than in primary AML cells (#39) cells‐bearing mice (Figure 6E, Table 3). Meanwhile, after administration for a week, we chose mouse randomly in each group of sample #29‐bearing mouse and killed them. We found that splenomegaly was obviously relieved after Baicalein treatment (Figure 6F). Engrafted leukemia cells were obviously infiltrated into bone marrow of primary AML cells (#29)‐bearing mice. Notably, huCD45^+^ sporadically distributed in the bone marrow of mice treated with Baicalein or VPA. In addition, either Baicalein or VPA treatment decreased the expression of HDAC‐1 in huCD45^+^ leukemia cells in bone marrow of primary AML cells (#29)‐bearing mice (Figure 6G). Furthermore, the proportion of huCD45^+^ cells in blood dramatically decreased in both Baicalein and VPA‐treated groups in #29 sample‐bearing mice (Figure 6H).

## 4 DISCUSSION

HDACs inhibition has been proved to show therapeutic effect through affecting proliferation, differentiation, and survival of AML cells. In the present study, we found that Baicalein inhibited enzyme activities of HDAC‐1/8 and induced degradation of HDAC‐1 protein. Meanwhile, we found that Baicalein inhibited proliferation and induced differentiation of U937, THP‐1, Kasumi‐1, and SKNO‐1 cell lines and primary AML cells derived from a cohort of AML patients. Notably, t(8; 21)q(22;22) AML cell lines and primary AML cells were more sensitive to Baicalein and the differentiation effect was much more outstanding than other cell lines. In inv(16) AML ME‐1 cells, the IC_50_ value of ME‐1 was 5.17 ± 1.11 µM at 96 h. However, in U937, THP‐1, Kasumi‐1, and SKNO‐1 cells, the IC_50_ values of those cells ranged from 13.73 ± 1.07 to 63.94 ± 1.32 µM after Baicalein treatment for 96 h, suggesting different mechanisms induced by Baicalein in ME‐1 cells and other AML cells.

In t(8;21)q(22;22) AML cells, HDAC‐1 is involved in pathogenesis mechanism induced by fusion protein AML1‐ETO produced by t(8; 21)q(22;22).^19^, ^55^, ^56^ We observed that HDAC‐1 degradation and C/EBPα upregulation in AML cells after treatment of Baicalein for 96 h when differentiation could be detected. Besides, during the treatment of Baicalein, we also found the AML1‐ETO degradation that was associated with the impairment of HSP90 chaperone function. Strategies to avoid resistance of HDAC inhibitors include employing combination therapies simultaneously targeting HDACs and HSP90.^57^ HSP90 was a molecular chaperone to stabilize longevity protein, and AML1‐ETO was one of client proteins of HSP90. Furthermore, HDAC‐1 has been reported to promote the deacetylation of HSP90 in the nucleus of human breast cancer cells.^58^ Moreover, HDAC‐1, HDAC6 and HDAC‐10 have been shown to regulate HSP90 chaperoning VEGF receptor proteins.^58^, ^59^ Baicalein was a natural product with broad spectrum of pharmacological functions and low toxicity. After treatment of Baicalein, the acetylation of HSP90 increased and the interaction of HSP90 and AML1‐ETO could be reduced. Results of immunoprecipitation and Duolink assay proved that the binding of AML1‐ETO and HSP90 was interrupted by Baicalein, which could be the key cause of degradation of AML1‐ETO.

As we know, LSCs contributed to the leukemogenesis, and maintained the relapse of AML.^60^ Recent research has shown that CM fusion protein disrupted p53 activity through aberrant posttranslational modification mediated by HDAC‐8, thus promoting CM‐associated LSCs transformation and maintenance.^24^ In our study, we found that Baicalein showed better inhibitory effects in inv(16) AML cells and induced the apoptosis of ME‐1 and primary AML cells with inv(16). In inv(16) AML, CM fusion protein recruits HDAC‐8 and p53 as a protein complex in which p53 acetylation is impaired by HDAC‐8.^24^ Therefore, we next explored the influence of Baicalein on CM‐recruited p53. Results showed that Baicalein increased the acetylation of CM‐bound p53, while the p53 level was slightly changed. Results of RT‐qPCR assay showed that downstream genes of p53 were activated, which was consistent with increased acetylation of p53. Subsequently, we investigated the effect of Baicalein on CD34^+^ inv(16) AML cells. Results showed that Baicalein induced a remarkable increase of apoptosis in the quiescent AML CD34^+^ population. Taken together, Baicalein restored p53 acetylation in inv(16) AML cells and diminished survival of AML CD34^+^ cells.

C/EBPα is a vital transcription factor in control of lineage‐specific gene expression in hematopoiesis that acts as a tumor suppressor in a number of malignancies.^61^ In several AML subtypes, C/EBPα expression is downregulated, resulting in a blockade of monocytic or granulocytic differentiation.^62^, ^63^ mRNA of C/EBPα can be translated from two different AUG codes, giving rise to two distinct isoforms, p42 and p30.^64^ The p30 informs lacks the transactivation domain 1, which is required for C/EBPα to interact with TATA box‐binding proteins and other transcription initiation factors. This truncated p30 isoform is known to inhibit p42 C/EBPα‐mediated transactivation of target genes.^65^, ^66^ The ratio of p42 to p30 reflects the differentiation level of AML cells, and differentiation inductors, such as ATRA, have been reported to increase the proportion of p42/p30 in THP‐1, U937, and COCL‐48.^67^, ^68^ Notably, in Baicalein‐induced differentiation effects in t(8; 21)q(22;22) AML cells, the level of p42 increased when p30 mildly decreased, which was different from a dramatic decrease of p30 in non‐CBF AML cell lines including THP‐1 and U937. As for the variance of C/EBPα regulation by Baicalein, we speculated that Baicalein exerted upregulation of either p42 or p30 via the degradation effect of AML1‐ETO fusion protein in AML with t(8; 21), but the influence by Baicalein in modulating p42/p30 ratio made the expression of p30 finally showed a slight change. In non‐CBF‐AML cells, Baicalein‐induced differentiation may be more likely to associate with regulation on p42/p30 ratio, which needs to be further studied.

HDACs inhibitors are generally capable of causing the upregulation of MDR1, then inducing a broad and pleiotropic drug resistance of AML cells by regulating multiple ABC transporter genes, which is associated to poor prognosis.^69^ Baicalein restored the acetylation of Histone via HDAC inhibition, though it did not affect the expression of HDAC inhibition‐associated ABC transporter genes. Recent research showed that Baicalein can inhibit TNF‐α‐induced NF‐κB activation and expression of NF‐κB‐regulated target gene. Meanwhile, inhibition on NF‐κB activity significantly decreased *MDR1* gene expression and drug resistance in HL‐60 cells, suggesting that Baicalein might be capable of suppressing *MDR1* gene expression via inhibiting NF‐κB signaling.^70^, ^71^ In addition, previous studies have shown an inhibitory effect of Baicalein on p‐gp expression and reversal of multidrug resistance in vivo and in vitro.^39^, ^72^, ^73^ Above all, the activities of Baicalein in suppressing *MDR1* gene and inhibiting p‐gp might be the cause of remaining unchanged HDACs‐inhibition‐associated ABC transporter genes.

Even though HDACs inhibition strategy has been known as a promising approach for the treatment of cancers, the clinical efficacy of HDAC inhibitors was limited by board ABC‐associated drug resistance in several cancers.^39^ Our study suggested that Baicalein showed HDAC‐1/8 inhibition effects and exerted outstanding anti‐leukemia effects in CBF‐AML, while no effects on the expression of HDAC inhibitor‐associated ABC transporter genes could be observed. In CBF‐AML, Baicalein showed a growth inhibition and differentiation induction in t(8; 21)q(22;22) AML cells and apoptosis effects in inv(16) AML cells, respectively. HDAC‐1 inhibition by Baicalein caused AML1‐ETO destabilization and expression of repressed genes that were associated to differentiation of t(8; 21)q(22;22) AML cells. Targeting HDAC‐8 resulted in an improvement of p53 acetylation and apoptosis of CD34^+^ cells with inv(16). This study demonstrated that Baicalein had a preferential inhibitory effect in CBF‐AML cells, suggesting the potential of Baicalein in developing into a novel agent for the treatment of AML.

## 5 CONCLUSION

These findings improved the understanding of the epigenetic regulation of Baicalein, showed that Baicalein inhibited the activity of HDAC‐1 and HDAC‐8 without promoting ABC transporter genes expression, and warrant therapeutic potential of Baicalein for CBF‐AML.

## ETHICS APPROVAL AND CONSENT TO PARTICIPATE

The animal study was carried out according to the regulations of the China Food and Drug Administration (CFDA) on Animal Care. All patients’ samples were collected after informed consent in accordance with the Declaration of Helsinki. The study was approved by the Ethics Committee of China Pharmaceutical University.

## AVAILABILITY OF DATA AND MATERIAL

The datasets used and/or analyzed during the current study are available from the corresponding author on reasonable request.

## CONFLICT OF INTEREST

The authors have declared that no competing interest exists

## AUTHOR CONTRIBUTIONS

X.Y. designed and performed research, analyzed data, and wrote the paper; H.L. performed research and analyzed data; P.H. performed research; Y.Q. and X.W. collected data and performed statistical analysis; M.Z., H.W., and Z.W. collected and analyzed data; J.X. provided the blood samples; and Q.G. and H.H. conceptualized the project and directed the experimental design and data analysis.

## Supporting information

Supporting Information.
