# Supplementary material for: Natural HDAC‐1/8 inhibitor baicalein exerts therapeutic effect in CBF‐AML
Source: Clin Transl Med. 2020 Aug 26;10(4):e154. doi: 10.1002/ctm2.154 (PMC7449246; doi:10.1002/ctm2.154)
Supplement: Supplementary file 1 — Supporting Information. [file CTM2-10-e154-s001.docx]

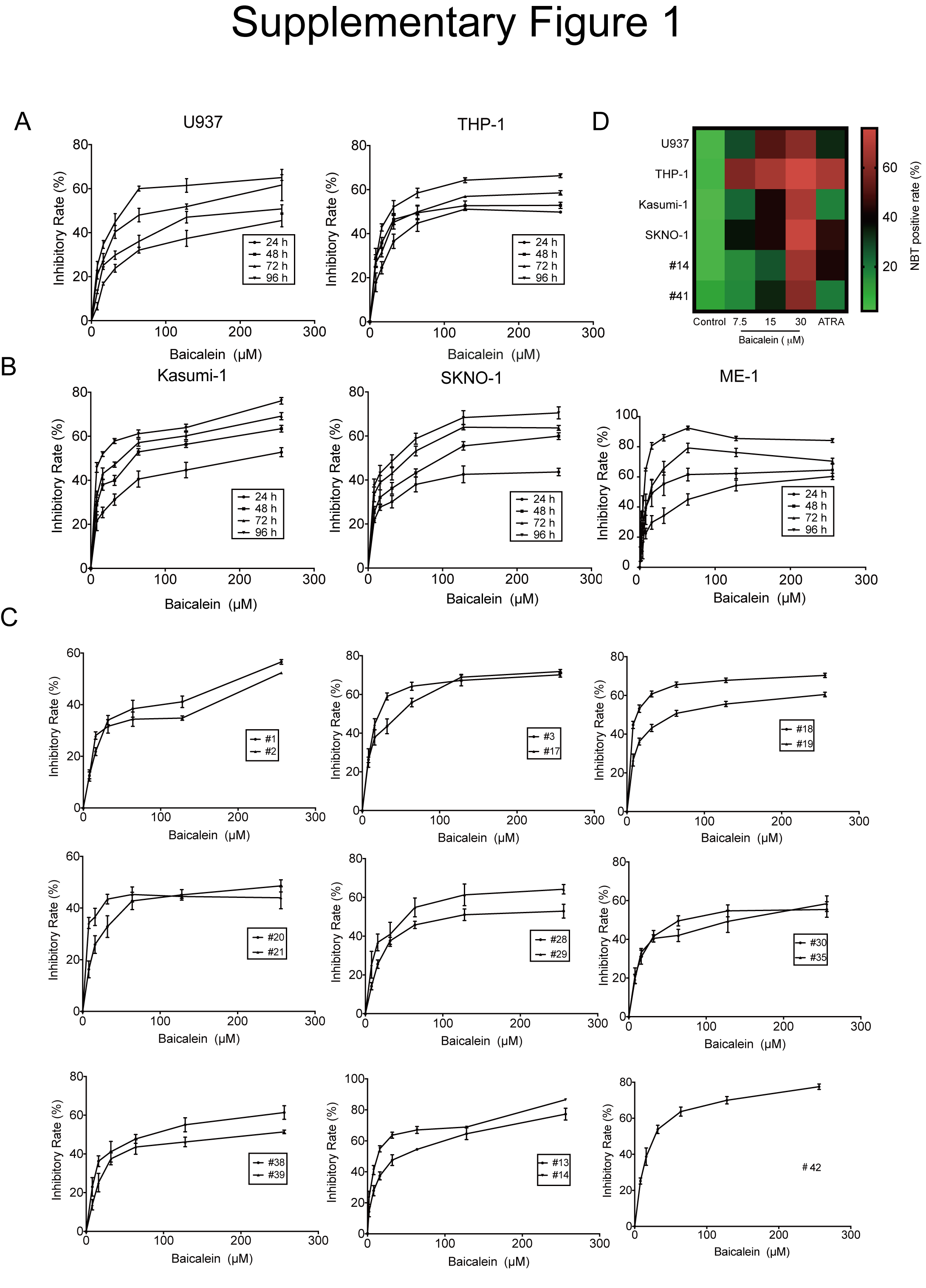


Supplementary Figure 1

(A-C) The growth inhibition effect of Baicalein (0-256 μM) on U937, THP-1, Kasumi-1, SKNO-1, ME-1 cells were assessed by CCK8 assay at 24, 48, 72 and 96 h. The growth inhibition effect of Baicalein(0-256 μM) on primary AML cells (#1, #2, #3, #13, #14, #17, #18, #19, #20, #21, #28, #29, #30, #35, #38, #39, and #42) were assessed by CCK8 assay at 96 h. Those data represent the mean ± SD of 3 different experiments.

(D) Detection of NBT-reduction activity of the AML cells (U937, THP-1, Kasumi-1, SKNO-1) and primary AML cells (#14, #41) after treatment with Baicalein (0, 7.5, 15 and 30 μM) and ATRA (1 μM) for 96 h. NBT-positive cells with purple-black color were counted, and the overall percentage was calculated based on 100 total cells per microscopic field and counting 5 times in each group.


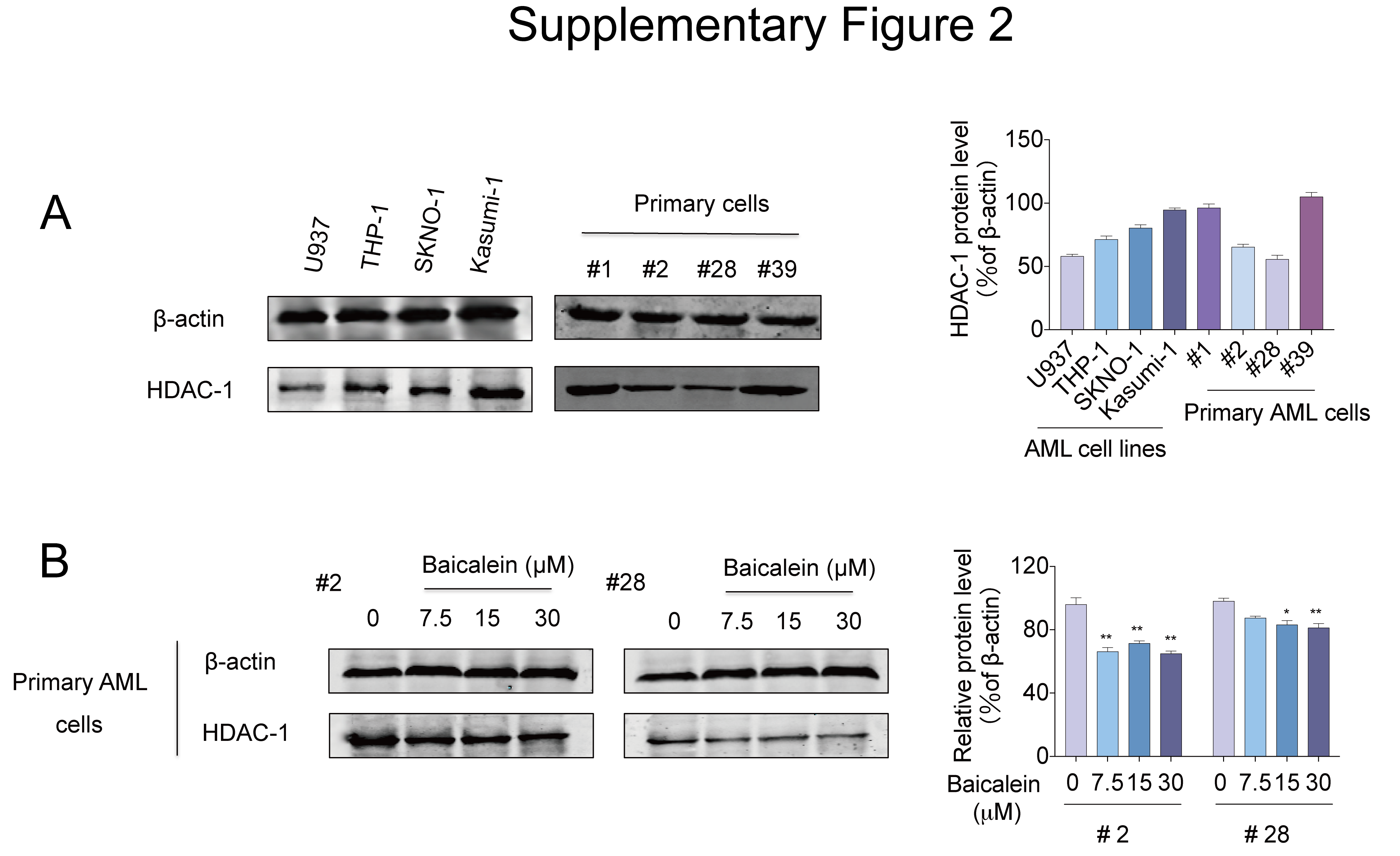


Supplementary Figure 2

(A) The background expression levels of HDAC-1 in U937, THP-1, SKNO-1, Kasumi-1 cell lines and primary AML cells (#1, #2, #28, and #39) were detected by western blot assay. β-actin were used as loading controls.

(B) Primary AML cells (#2, #28) were treated with Baicalein (0, 7.5, 15 and 30 μM) for 96 h. Expression levels of HDAC-1 were analyzed by western blot. β-actin were used as loading controls.

Those data represent the mean ± SD of 3 different experiments. Asterisks denote statistically significant *,P < 0.05; **, P < 0.01; differences compared with controls by one-way ANOVA.


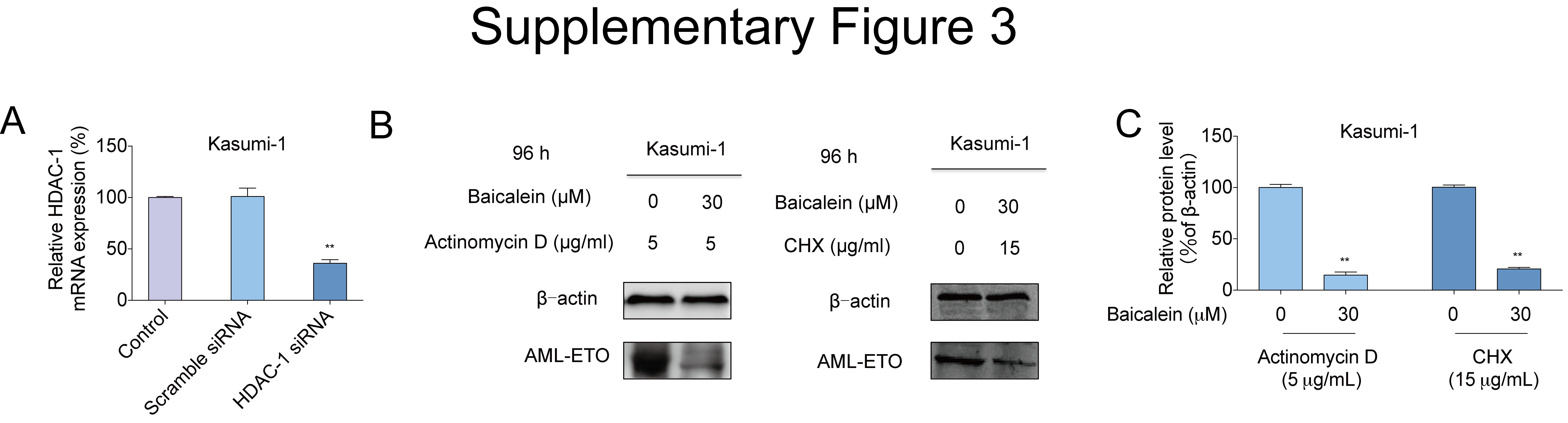


Supplementary Figure 3

(A) The Kasumi-1 cells were transfected with *HDAC-1* siRNA for 48 h, and the efficacy of *HDAC-1* siRNA transfection was monitored by RT-qPCR.

(B, C) Kasumi-1 cells were treated with 15 μg/mL CHX or 5 μg/mL Actinomycin D, and/or 30 μM Baicalein for 96 h. Western blot was performed to detect the expression of AML1-ETO. β-actin were used as loading controls.

The data represent the mean 3 different experiments. Asterisks denote statistically significant *, P < 0.05; **, P < 0.01; differences compared with controls by one-way ANOVA.


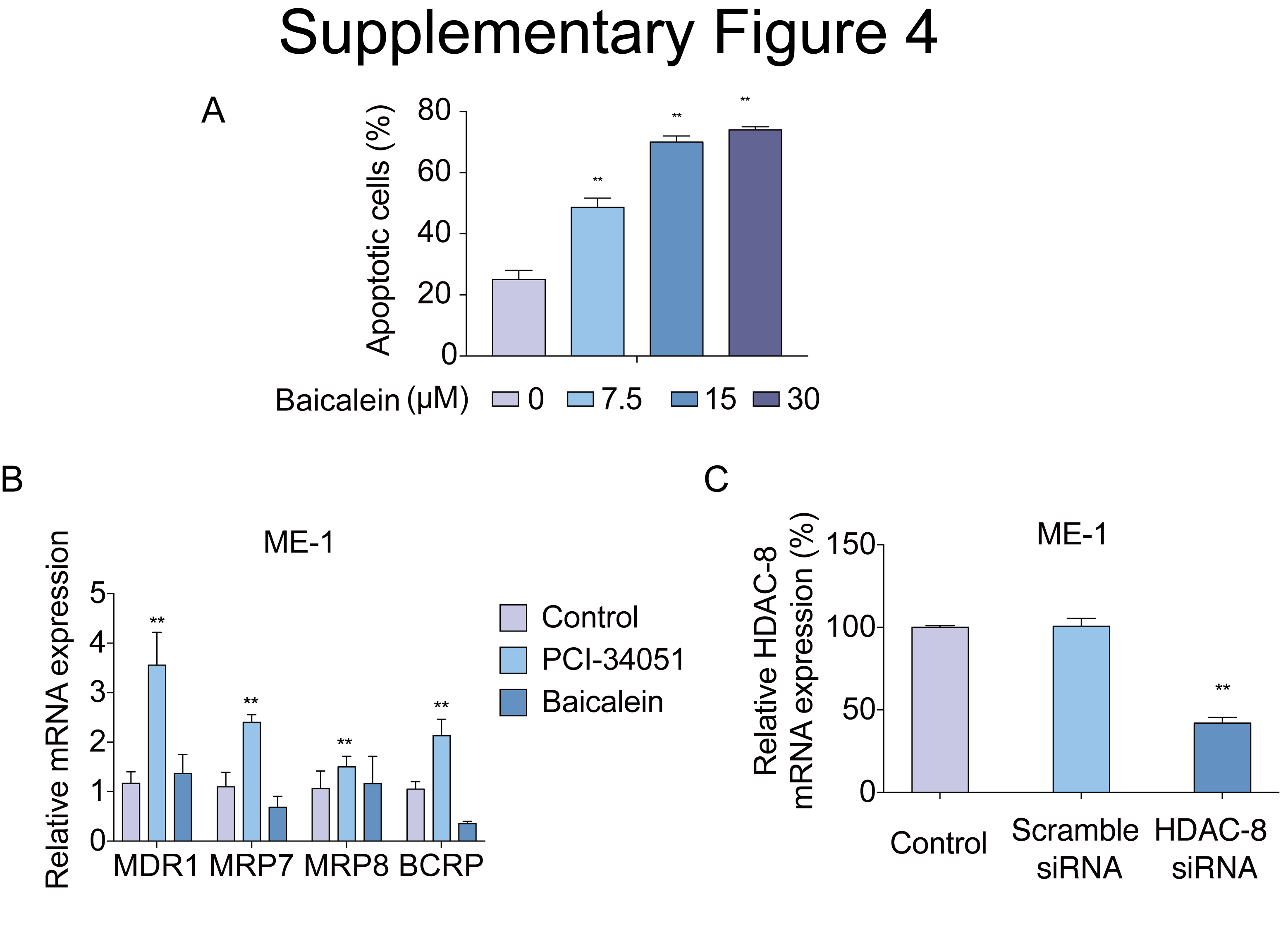


Supplementary Figure 4

(A) Apoptosis effects of Baicalein on primary AML cells (# 42). Cells were treated with Baicalein (0, 7.5, 15 and 30 μM) for 48 h. Results were analyzed by flow cytometry.

(B) ME-1 cells were treated with 40 μM PCI-34051 or 30 μM Baicalein for 96 h. Effects of HDAC inhibitors on *MDR1*, *MRP7*, *MRP8* and *BCRP* expression were analyzed by RT-qPCR.

(C) The ME-1 cells were transfected with *HDAC-8* siRNA for 48 h, and the efficacy of HDAC-8 siRNA transfection was monitored by RT-qPCR.

The data represent the mean 3 different experiments. Asterisks denote statistically significant *, P < 0.05; **, P < 0.01; differences compared with controls by one-way ANOVA.
